# Intravenous immunoglobulin mediates anti-inflammatory effects in peripheral blood mononuclear cells by inducing autophagy

**DOI:** 10.1038/s41419-020-2249-y

**Published:** 2020-01-23

**Authors:** Mrinmoy Das, Anupama Karnam, Emmanuel Stephen-Victor, Laurent Gilardin, Bharat Bhatt, Varun Kumar Sharma, Naresh Rambabu, Veerupaxagouda Patil, Maxime Lecerf, Fabian Käsermann, Patrick Bruneval, Kithiganahalli Narayanaswamy Balaji, Olivier Benveniste, Srini V. Kaveri, Jagadeesh Bayry

**Affiliations:** 1 Institut National de la Santé et de la Recherche Médicale; Centre de Recherche des Cordeliers, Equipe- Immunopathologie et Immunointervention Thérapeutique, Sorbonne Université, 75006 Paris, France; 2 Département de Médecine Interne et Immunologie Clinique, Hôpital Pitié-Salpêtrière, AP-HP, 75013 Paris, France; 3 Department of Microbiology and Cell Biology, Indian Institute of Science, Bangalore, 560012 India; 4 Université Paris Descartes, Sorbonne Paris Cité, 75006 Paris, France; 5 CSL Behring, Research, CSL Biologics Research Center, 3014 Bern, Switzerland; 6 Service d’anatomie pathologique, Hôpital Européen Georges Pompidou, 75015 Paris, France; 7 Institut National de la Santé et de la Recherche Médicale Unité 974, Sorbonne Université, 75013 Paris, France

**Keywords:** Autoimmunity, Immunotherapy, Translational research

## Abstract

Autophagy plays an important role in the regulation of autoimmune and autoinflammatory responses of the immune cells. Defective autophagy process is associated with various autoimmune and inflammatory diseases. Moreover, in many of these diseases, the therapeutic use of normal immunoglobulin G or intravenous immunoglobulin (IVIG), a pooled normal IgG preparation, is well documented. Therefore, we explored if IVIG immunotherapy exerts therapeutic benefits via induction of autophagy in the immune cells. Here we show that IVIG induces autophagy in peripheral blood mononuclear cells (PBMCs). Further dissection of this process revealed that IVIG-induced autophagy is restricted to inflammatory cells like monocytes, dendritic cells, and M1 macrophages but not in cells associated with Th2 immune response like M2 macrophages. IVIG induces autophagy by activating AMP-dependent protein kinase, beclin-1, class III phosphoinositide 3-kinase and p38 mitogen-activated protein kinase and by inhibiting mammalian target of rapamycin. Mechanistically, IVIG-induced autophagy is F(ab′)_2_-dependent but sialylation independent, and requires endocytosis of IgG by innate cells. Inhibition of autophagy compromised the ability of IVIG to suppress the inflammatory cytokines in innate immune cells. Moreover, IVIG therapy in inflammatory myopathies such as dermatomyositis, antisynthetase syndrome and immune-mediated necrotizing myopathy induced autophagy in PBMCs and reduced inflammatory cytokines in the circulation, thus validating the translational importance of these results. Our data provide insight on how circulating normal immunoglobulins maintain immune homeostasis and explain in part the mechanism by which IVIG therapy benefits patients with autoimmune and inflammatory diseases.

## Background

Recent reports show that macroautophagy, hereafter referred to as autophagy, plays an important role in the regulation of autoimmune and autoinflammatory responses. Autophagy involves lysosomal degradation of unnecessary or dysfunctional cellular components, cytotoxic mediators and misfolded proteins. During this process, targeted cytoplasmic constituents are isolated from the rest of the cell within a double-membraned vesicle known as an autophagosome. The autophagosome then fuses with a lysosome and its cargo is degraded and recycled^1,2^. A series of autophagy-related proteins (ATG) and several kinases play an important role in the regulation of autophagy. While mammalian target of rapamycin (mTOR) and AKT negatively regulate autophagy process, kinases such as phosphoinositide 3-kinase (PI3K) class III, AMP-dependent protein kinase (AMPK), extracellular signal-regulated kinase (ERK) and Jun N-terminal kinase (JNK) positively regulate components of autophagy machinery. p38 mitogen-activated protein kinase (p38 MAPK) has a dual role in regulating autophagy^3^.

Autophagy was initially investigated as a process involved in the “cell survival” and nowadays is recognized as fundamental in the physiology of eukaryotic cells. Autophagy also plays a fundamental role in the regulation of innate and adaptive immune responses^4^, lymphocyte differentiation, survival and homeostasis^4,5^. Several reports show the role of autophagy in the regulation of autoimmune and inflammatory pathologies including systemic lupus erythematosus, inflammatory bowel diseases, rheumatoid arthritis, psoriasis, multiple sclerosis and myositis^6–15^ and hence autophagy represents potential target to treat autoimmune and inflammatory diseases^16^. Moreover, in many of these diseases, the therapeutic use of intravenous and subcutaneous immunoglobulin (IVIG/SCIG), the normal human IgG preparations obtained from the pools of plasma of several thousand healthy donors is well documented^17–20^.

In addition to its use in primary and secondary immune deficiencies, IVIG is increasingly used for the treatment of large number of autoimmune and inflammatory diseases^17–20^. Data from various labs have demonstrated that IVIG exerts immune homeostasis by suppressing the activation of innate and adaptive immune compartments, inhibiting the inflammatory mediators and enhancing the anti-inflammatory processes^21–24^. In view of common important role played by autophagy and IVIG in the regulation of autoimmune and inflammatory responses, we aimed to explore if IVIG immunotherapy exerts immune homeostasis and therapeutic benefits via induction of autophagy.

## Results

### IVIG induces autophagy in peripheral blood mononuclear cells of healthy donors

**Fig. 1:**
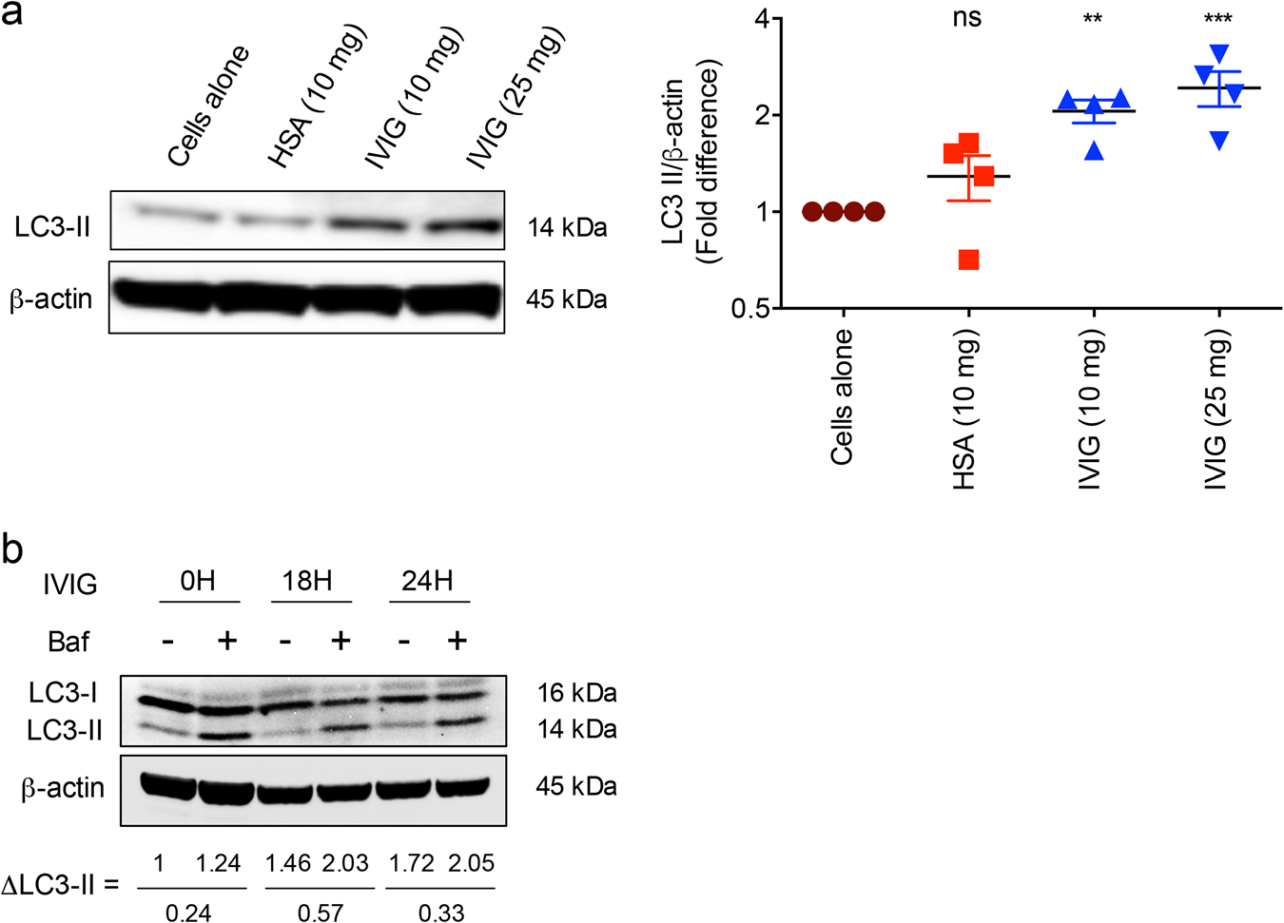
IVIG induces autophagy in peripheral blood mononuclear cells. **a** PBMCs from healthy donors were cultured with IVIG (10 or 25 mg/ml) or HSA for 24 h. Cells were treated with bafilomycin (Baf) for last 45 min. Levels of LC3-II and the fold changes in LC3-II levels (mean ± SEM, *n* = 4 donors) based on the densitometry analysis of western blots are presented. **P < 0.01; ***P < 0.001; ns not significant; by one-way ANOVA with Dunnett’s multiple comparison test. **b** Induction of autophagy flux by IVIG. PBMCs of healthy donors were cultured with IVIG (25 mg/ml) for 0, 18, or 24 h. At the end of treatment, cells were treated with or without Baf for 45 min. Autophagy flux (∆LC3-II) was quantified by subtracting the band intensity of LC3-II in the presence of Baf from that of absence of Baf. Representative blot of two different donors is shown.

In order to investigate whether IVIG induces autophagy, we first resorted to peripheral blood mononuclear cells (PBMCs) of healthy donors. PBMCs were cultured with different doses of IVIG (10 and 25 mg/ml) for 24 h and analyzed by western blot for the level of LC3-II, one of the well-characterized markers of autophagy^25^. Untreated PBMCs showed minimal level of LC3-II and its expression was not altered by equimolar concentrations of human serum albumin (HSA), an irrelevant protein control for IVIG. However, treatment of PBMCs with IVIG led to significantly enhanced level of LC3-II (Fig. 1a). Further, the effect of IVIG on autophagy was dose-dependent. High level of LC3-II was observed at 25 mg/ml, a concentration of IVIG reaches in the circulation of autoimmune patients immediately following therapy. As LC3-II was also enhanced in cells treated with 10 mg concentration of IVIG, which correspond to IgG in the circulation of healthy individuals, this suggests that circulating normal IgG induces autophagy under physiological conditions. Induction of autophagy by IVIG was also confirmed through the analyses of autophagy flux (Fig. 1b).

### IVIG induces autophagy in monocytes, dendritic cells, and M1 macrophages, but not in M2 macrophages

**Fig. 2:**
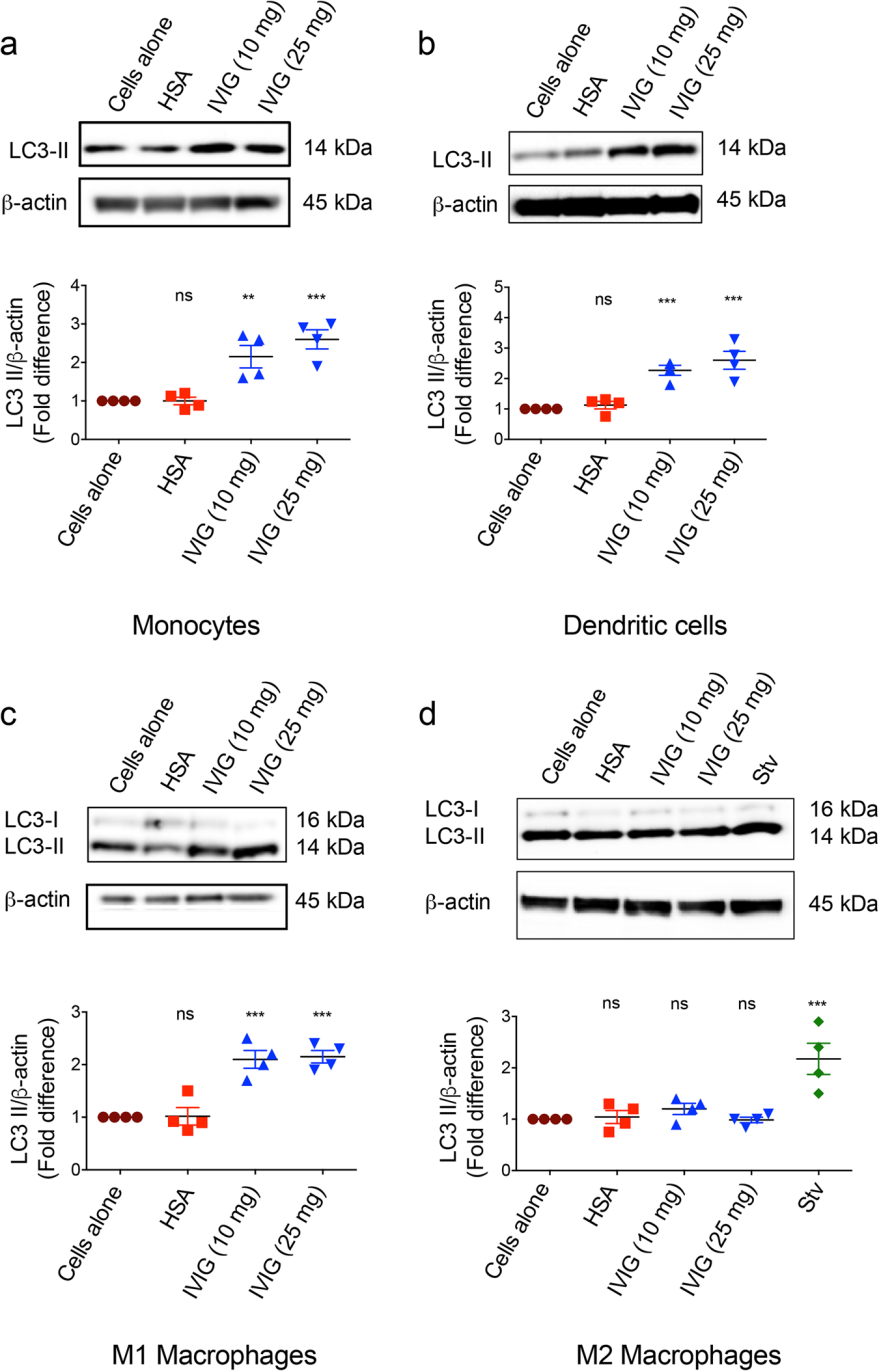
IVIG induces autophagy in monocytes, dendritic cells and M1 macrophages but not in M2 macrophages. **a**–**d** Peripheral blood monocytes (**a**), monocyte-derived DCs (**b**), M1 macrophages (**c**) and M2 macrophages (**d**) were cultured with IVIG for 24 h. Levels of LC3-II and fold changes in the LC3-II levels (mean ± SEM, *n* = 4 donors) are presented. Starvation condition (Stv) was used as a positive control for autophagy. Images are cropped for the presentation. **P < 0.01; ***P < 0.001; ns not significant; by one-way ANOVA with Dunnett’s multiple comparison test.

As innate immune cells are central for initiating and propagating the immune responses, we then explored if the ability of IVIG to induce autophagy is universal for all types of innate immune cells. To address this question, we exposed peripheral blood monocytes, and monocyte-derived dendritic cells (DCs), M1 macrophages and M2 macrophages to different doses of IVIG (10 and 25 mg/ml). We found that among innate immune cells, IVIG-induced autophagy was restricted to inflammatory cells like monocytes, DCs and M1 macrophages but not in cells associated with Th2 immune response like M2 macrophages (Fig. 2a–d). We further confirm that inability of IVIG to induce autophagy in M2 macrophages is not due to technical errors or defects in the cells to undergo autophagy as starvation condition could induce autophagy in M2 cells (Fig. 2d). These results thus suggest that IVIG exhibits selective ability to induce autophagy and that all immune cells are not susceptible to IVIG-induced autophagy.

**Fig. 3:**
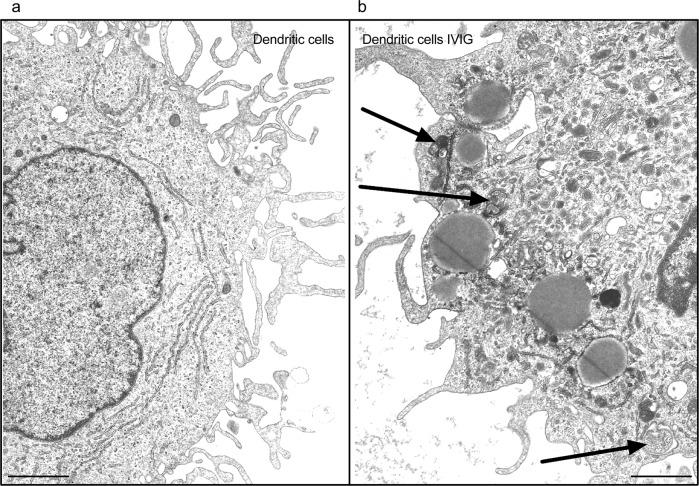
Transmission electron microscopic analysis of autophagic organelles induced by IVIG in dendritic cells. **a**, **b** Lipopolysaccharide-stimulated monocyte-derived human DCs were cultured for 24 h in the absence (**a**) or presence of IVIG (**b**). The cells were then processed for the transmission electron microscopy to visualize autophagic organelles. Arrows show autophagosomes. (scale bar = 100 nm).

In order to obtain additional evidence on the IVIG-induced autophagy, we performed transmission electron microscopy of lipopolysaccharide-stimulated DCs to visualize autophagic organelles. While control DCs did not show autophagosome structures in the cytoplasm, IVIG-treated DCs on the other hand displayed double-membraned autophagosomes with undigested or partially degraded cytoplasmic contents (including endoplasmic reticulum) (Fig. 3a, b). These data thus validate that IVIG induces autophagy in inflammatory immune cells.

### IVIG-induced autophagy is associated with induction and activation of AMPK, beclin-1, class III PI3K and p38MAPK, and inhibition of mTOR activation

**Fig. 4:**
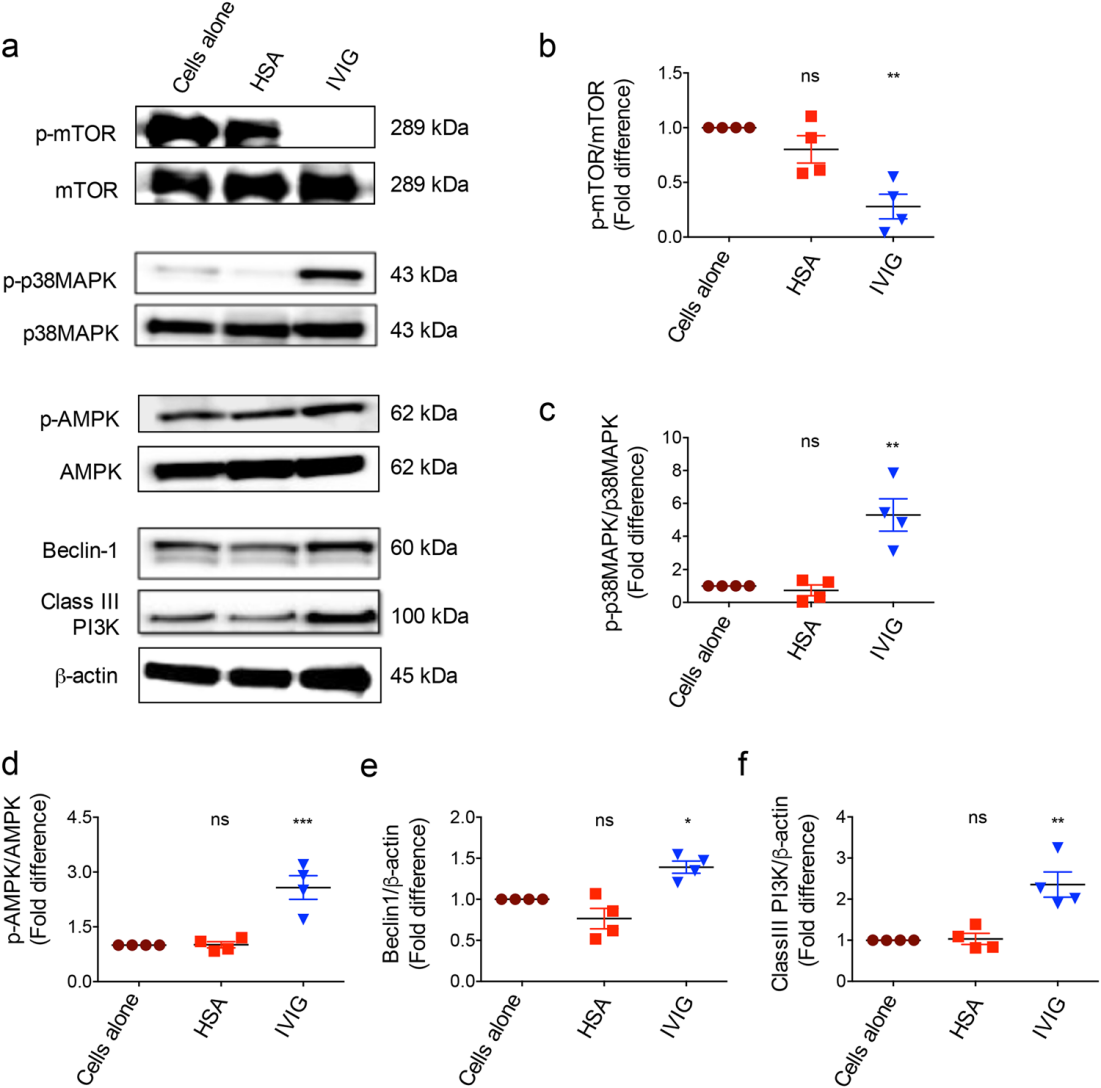
IVIG-induced autophagy in monocytes is associated with induction of class III PI3K, beclin-1, p-AMPK and p-p38MAPK and inhibition of p-mTOR. **a** Monocytes were cultured with IVIG (25 mg/ml) or HSA for 24 h. Expression of p-mTOR, mTOR, p-p38MAPK, p38MAPK, p-AMPK, AMPK, beclin-1 and class III PI3K was analyzed by western blot. Representative blots of four experiments by using monocytes from different donors are shown. **b**–**f** Densitometry analysis of fold changes (mean ± SEM, *n* = 4) in the expression of p-mTOR (**b**), p-p38MAPK (**c**), p-AMPK (**d**), beclin-1 (**e**), and class III PI3K (**f**). *P < 0.05; **P < 0.01; ***P < 0.001; ns not significant; as determined by one-way ANOVA Dunnett’s multiple comparison test.

Several kinases and signaling molecules play an important role in the regulation of autophagy^1,3^. Therefore, to gain further insight on the signaling molecules implicated in IVIG-induced autophagy, we probed the expression and activation status of various signaling molecules that are critical for the formation of autophagosome. All experiments for the dissection of mechanisms were performed in monocytes. We found that IVIG significantly reduced the phosphorylated form of mTOR without modulating total mTOR expression (Fig. 4a, b). On the other hand, IVIG induced beclin-1 and class III PI3K and significantly enhanced the phosphorylation of p38 MAPK and AMPK (Fig. 4a, c–f).

### Inhibition of Class III PI3K, AMPK and p38 MAPK activation leads to abrogation of IVIG-induced autophagy

**Fig. 5:**
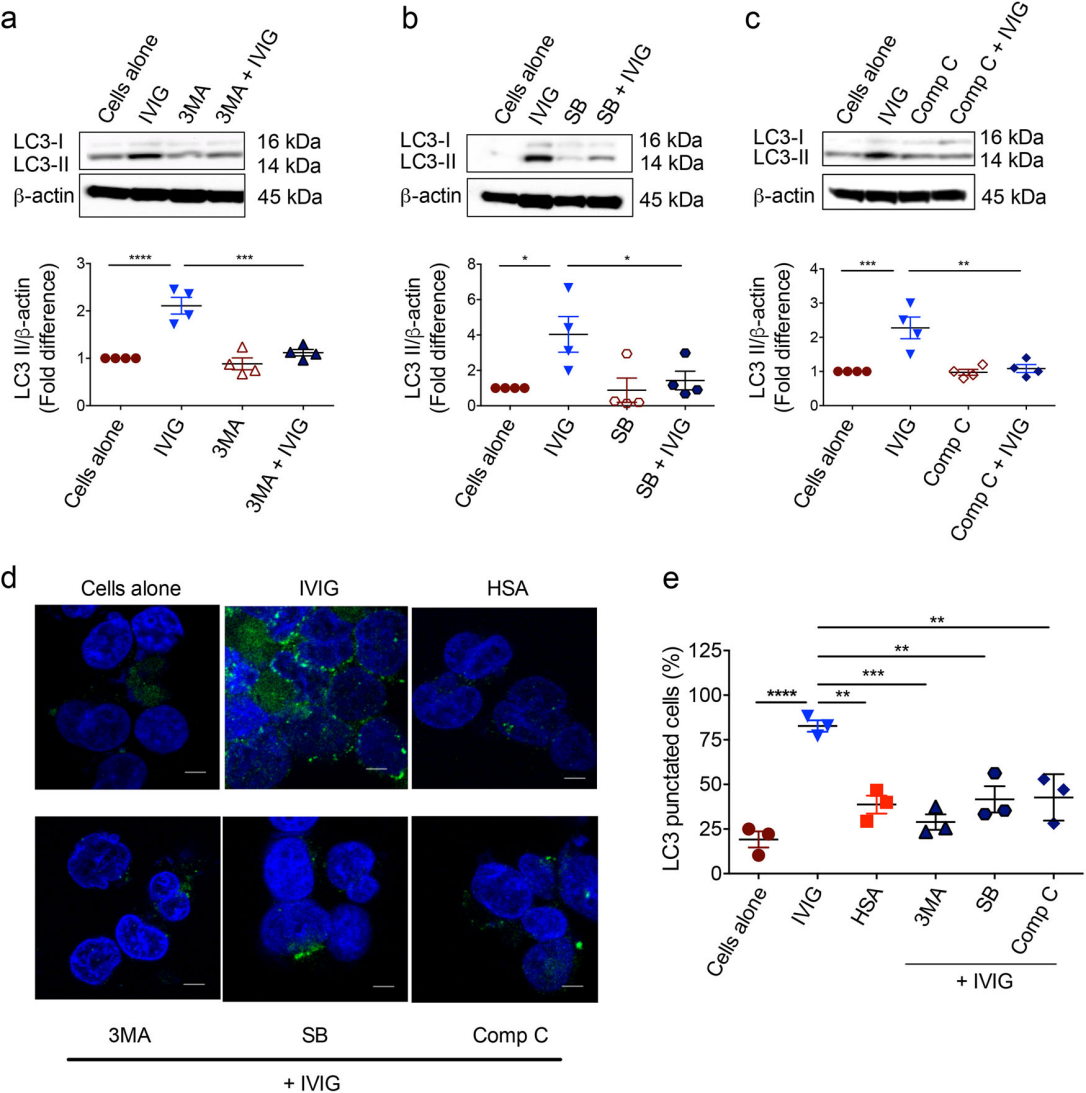
Inhibition of Class III PI3K, p38MAPK and AMPK kinases abrogates IVIG-induced autophagy in monocytes. **a**–**c** Peripheral blood monocytes were pretreated with **a** Class III PI3K inhibitor: 3MA, **b** p38MAPK inhibitor: SB202190 and **c** AMPK inhibitor: Compound C, followed by IVIG treatment for 24 h. Levels of LC3-II by western blot and densitometry analyses of fold changes (mean ± SEM; *n* = 4 donors) are presented. Images are cropped for the presentation. **d**, **e** THP-1 cells were either cultured alone or treated with IVIG or HSA for 24 h. In other set of experiments, cells were pretreated with respective inhibitors followed by IVIG treatment for 24 h. The cells were then stained with anti-MAP1LC3I/II primary antibody followed by Alexa Fluor® 488-conjugated secondary antibody. Nuclei were stained with DAPI. Representative fluorescent images and percent (mean ± SEM; *n* = 3) of LC3-punctated cells are shown. Bars indicate magnifications (×63. Scale bars = 5 µm). *P < 0.05; **P < 0.01; ***P < 0.001; ****P < 0.0001; as determined by one-way ANOVA Dunnett’s (**a**–**c**) or Tukey’s multiple comparison test (**e**).

To validate the role of Class III PI3K, AMPK and p38 MAPK kinases in IVIG-induced autophagy, monocytes were pretreated with different pharmaceutical inhibitors, i.e., 3-methyladenine (3MA, for class III PI3K), SB202190 (for p38 MAPK) and compound C (for AMPK) followed by culture with IVIG for 24 h. In line with the data on activation of these kinases by IVIG (Fig. 4), inhibition of Class III PI3K, AMPK and p38 MAPK kinases led to the abrogation of IVIG-induced autophagy as analyzed by LC3-II protein level by western blot (Fig. 5a–c).

To further authenticate IVIG-induced autophagy, we resorted to fluorescence microscopy to visualize LC3 puncta in IVIG-treated THP-1 cells, a human monocytic cell line. We found significantly higher characteristic LC3 puncta formation in the cells upon treatment with IVIG (Fig. 5d, e), thus confirming the western blot results. Moreover, pretreatment of THP-1 cells with 3MA, SB202190 and compound C significantly abolished IVIG-induced LC3 puncta formation (Fig. 5d, e). These results thus demonstrate that IVIG induces autophagy through activation of class III PI3K, p38 MAPK and AMPK kinases.

### Induction of autophagy by IVIG is F(ab′)_2_-dependent but sialylation independent

**Fig. 6:**
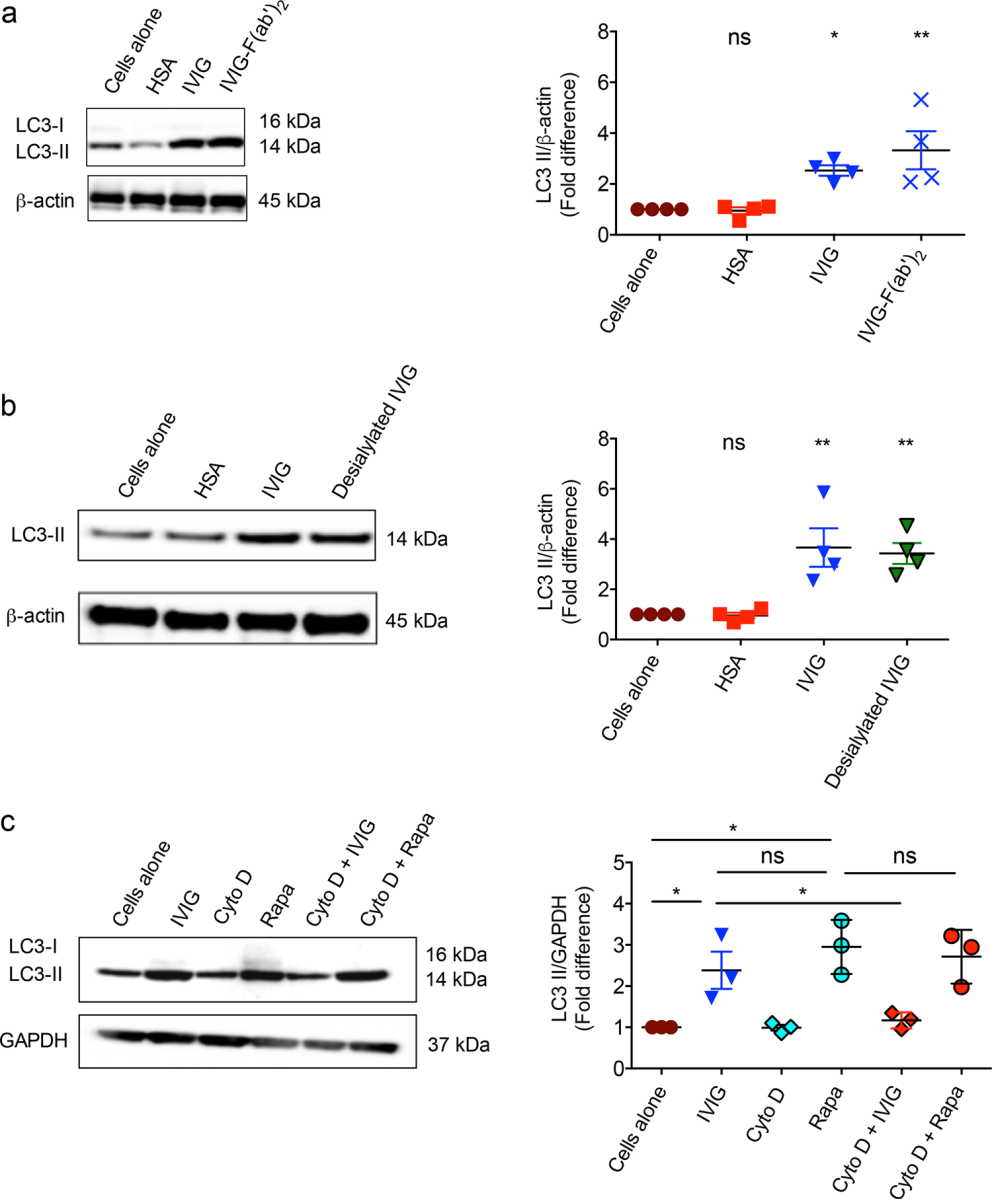
IVIG-induced autophagy is F(ab′)_2_-dependent but sialylation-independent, and requires endocytosis of IgG. **a**, **b** Monocytes were cultured with IVIG (25 mg/ml), equimolar concentration of F(ab′)_2_ or desialylated IVIG for 24 h. Levels of LC3-II were analyzed by western blot. Representative blots of four different donors and fold changes in the LC3-II levels based on densitometry analysis of western blots (mean ± SEM, *n* = 4 donors) are presented. Images are cropped for the presentation. **c** Monocytes were pretreated with cytochalasin D (Cyto D) followed by IVIG or rapamycin (Rapa) treatment for 24 h. Levels of LC3-II by western blot and densitometry analysis of fold changes (mean ± SEM; *n* = 3 donors) are shown. *P < 0.05; **P < 0.01; ns not significant; as determined by one-way ANOVA with Dunnett’s multiple comparison test.

IgG consists of Fc and Fab regions. While some mechanisms of IVIG were dependent on Fc-fragments, other mechanisms were mediated via F(ab′)_2_ fragments^21^. Therefore, we investigated the role of F(ab′)_2_ fragments to dissect the mechanisms of IVIG-induced autophagy. Monocytes were cultured with equimolar concentrations of either IVIG or F(ab′)_2_ fragments for 24 h. Interestingly, F(ab′)_2_ fragments of IVIG significantly enhanced the levels of LC3-II (Fig. 6a). These results thus demonstrate that autophagy induction by IVIG is F(ab′)_2_-dependent.

Sialylation of IgG has been proposed to mediate several anti-inflammatory actions of IVIG though other reports also demonstrate sialylation-independent anti-inflammatory effects^26–33^. Therefore, we investigated if IVIG-induced autophagy is dependent on its sialylation content. However, we found that desialylation of IgG had no repercussion on IVIG-induced autophagy (Fig. 6b).

### Endocytosis of IgG is mandatory for the IVIG-mediated autophagy in innate immune cells

Numerous reports have shown that innate immune cells internalize IVIG by endocytosis^28,34,35^. We therefore investigated whether signaling cascades induced by IVIG upon binding of IgG to the surface of innate immune cells is sufficient for inducing autophagy or it requires endocytosis of IgG. To probe this hypothesis, monocytes were pretreated with cytochalasin D, an inhibitor of endocytosis followed by culture with IVIG for 24 h. As shown in Fig. 6c, cytochalasin D treatment abrogated IVIG-induced autophagy indicating that endocytosis of IVIG is mandatory for the induction of autophagy in innate cells like monocytes.

To confirm that loss of IVIG-induced autophagy upon cytochalasin D treatment was not due to its adverse effects on the autophagy machinery itself, we treated monocytes with rapamycin either alone or in combination with cytochalasin D. We found that rapamycin-induced autophagy was not affected by cytochalasin D (Fig. 6c), thus confirming that cytochalasin D specifically inhibited IVIG-induced autophagy.

### Induction of autophagy is critical for the anti-inflammatory actions of IVIG

**Fig. 7:**
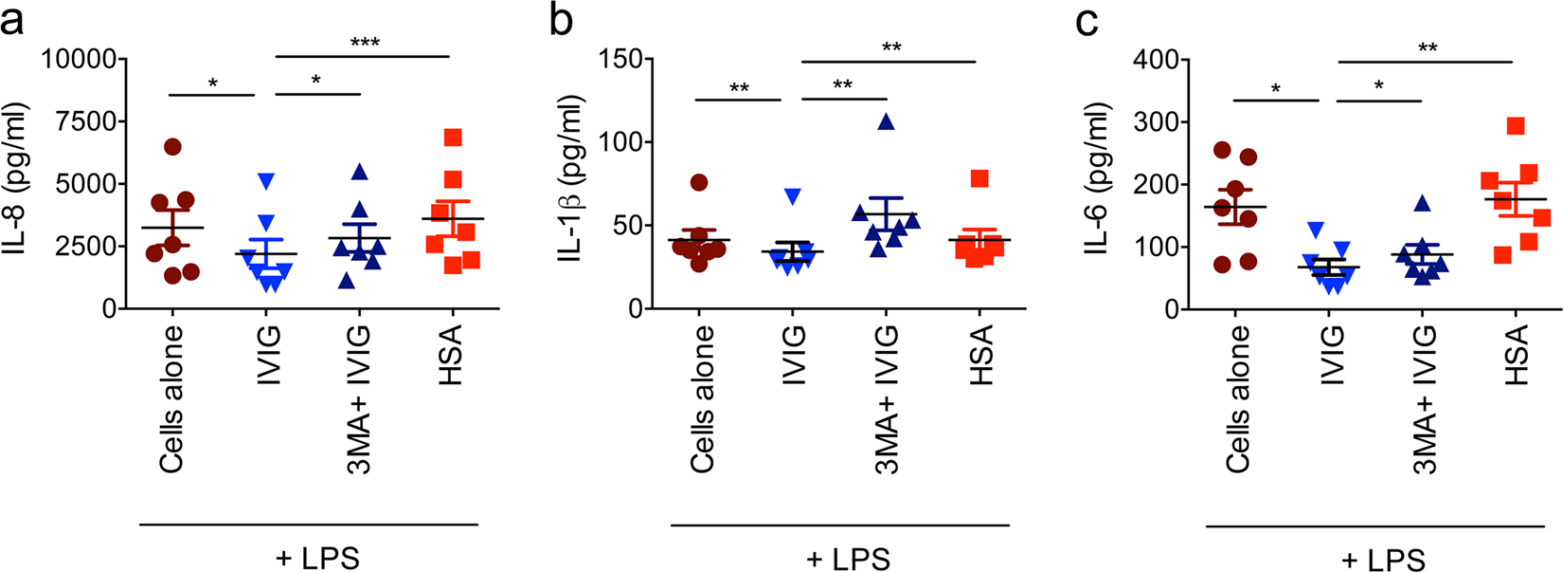
Induction of autophagy is critical for the anti-inflammatory actions of IVIG. LPS-stimulated DCs were pretreated with 3MA followed by culture with IVIG (25 mg/ml) for 24 h. Amounts of IL-8, IL-1β and IL-6 (mean ± SEM; *n* = 7 donors) in the cell-free supernatants were analyzed by ELISA. *P < 0.05; **P < 0.01; ***P < 0.001; as determined by one-way ANOVA with Tukey’s multiple comparison test.

After demonstrating that IVIG induces autophagy in various innate immune cells, we investigated if this process is critical for the anti-inflammatory actions of IVIG. By using lipopolysaccharide-stimulated human monocyte-derived DCs as a model, we found that the ability of IVIG to suppress the production of inflammatory cytokines like IL-8, IL-1β and IL-6 were compromised when autophagy in DCs was inhibited through 3MA (Fig. 7a–c)^36^. These data thus show that induction of autophagy is integral to the anti-inflammatory mechanisms of IVIG.

### IVIG therapy induces autophagy in autoimmune patients

**Fig. 8:**
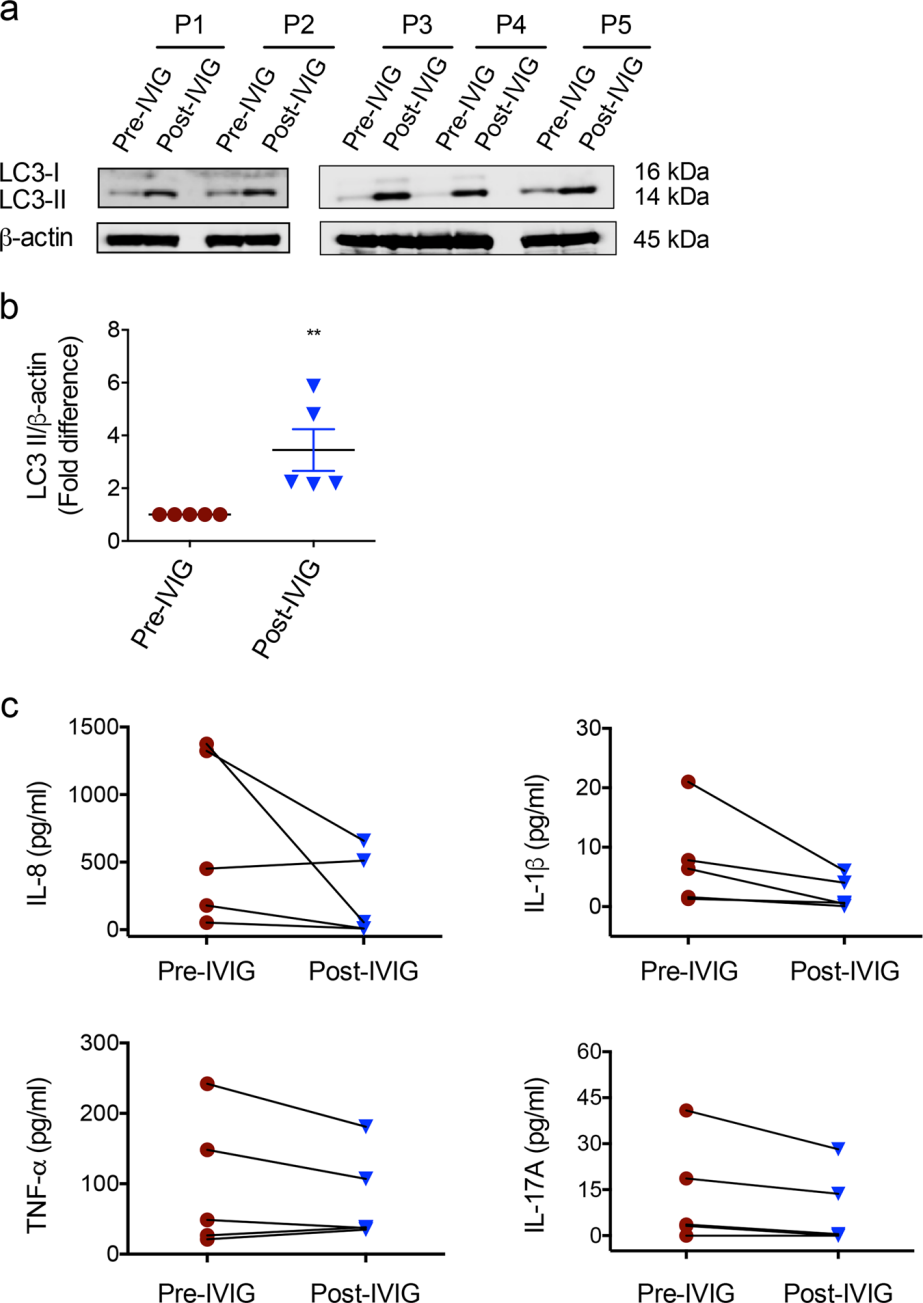
IVIG therapy in inflammatory myopathy patients induces autophagy. **a** Levels of LC3-II in PBMC of myopathy patients before (pre-IVIG) and post-IVIG therapy as analyzed by western blot (five patients, P1−P5). **b** Fold changes in the LC3-II levels based on the densitometry analysis of western blots (mean ± SEM, *n* = 5 patients) **P < 0.01 two-tailed Mann–Whitney test. **c** Changes in the levels (pg/ml) of inflammatory cytokines (IL-8, TNF-α, IL-1β and IL-17A) in the plasma of myopathy patients following IVIG therapy.

Indispensability of autophagy in mediating anti-inflammatory actions of IVIG raised an interesting perspective of whether this immunotherapy in autoimmune patients also induces autophagy. Earlier studies have indicated that IVIG therapy benefits patients with inflammatory myopathies^17–19^. Therefore, we investigated if IVIG therapy induces autophagy in these patients. PBMCs from the paired (pre- and post-IVIG therapy) blood samples of patients were subjected to western blot for the analysis of LC3-II. Prior to IVIG therapy, patients’ PBMCs showed minimal level of LC3-II. However, IVIG therapy led to significantly enhanced levels of LC3-II in the patients’ PBMC (Fig. 8a, b), indicating that IVIG treatment induces autophagy in autoimmune patients thus providing translation insight on our results.

To corroborate these results, we analyzed several inflammatory cytokines like IL-8, TNF-α, IL-1β and IL-17A in the plasma of these inflammatory myopathy patients following IVIG therapy. In line with the well-known anti-inflammatory effects of IVIG demonstrated in various pathologies, IVIG reduced the inflammatory cytokines in myopathy patients (Fig. 8c).

## Discussion

Immunoglobulins are classically known for their protective role against infections. However, several lines of evidence also suggest that immunoglobulins are critical for mediating immune homeostasis by clearing apoptotic, altered and senescent cells, protecting the mucosal layers, and hence blunting the inflammation and autoimmune responses^37–39^. Additional functions of circulating immunoglobulins and in particular IgG in immune homeostasis were comprehended through the therapeutic use of IVIG. As autophagy plays an important role in maintaining “healthy” status of the cell by clearing damaged organelles, cytotoxic molecules and misfolded proteins, we hypothesized that circulating normal IgG and hence IVIG exerts immune homeostasis via autophagy.

Several reports have demonstrated that cytokines play a key role in the regulation of autophagy in immune cells. Thus, proinflammatory cytokines including IFN-γ, TNF-α, IL-1 and IL-23 induce autophagy^40^, while Th2 and regulatory cytokines including IL-4, IL-13 and IL-10 are inhibitory^41,42^. Here, by using IVIG we provide compelling evidence that in addition to cytokines, the immunoglobulins that are normal constituents of a body potently regulate autophagy. As immunoglobulin deficiency is associated with high incidence of autoimmune and inflammatory diseases^43^, and that autophagy plays an important role in the regulation of autoimmune and autoinflammatory responses, our data suggest that immunoglobulins link autophagy to maintain immune homeostasis and to prevent immune aggression.

Autophagy is controlled by products of numerous Atg and several macromolecular signaling complexes including mTOR^3^. Inhibition of mTOR is essential for the initiation of autophagy and to allow the translocation of a complex containing Atg1/unc-51-like kinase1/2, Atg13, FIP200 and Atg101 from cytosol to the endoplasmic reticulum^1^. This leads to the recruitment of type III PI3K and VPS34 in a complex with other proteins, including beclin-1 to the developing autophagosome^1,3^. Rapamycin that inhibits mTOR and enhances autophagy has been successfully used in the treatment of rheumatoid arthritis^44,45^. In lupus-prone mice, rapamycin treatment prevents development of nephritis, inhibits lymphoproliferation, reduces autoantibody production and enhances the survival of mice^46^. Recent report in an experimental model also showed that preventive and curative treatment with rapamycin decreases the severity of myositis^47^. In view of these data from rapamycin and well-demonstrated beneficial effects of IVIG therapy in inflammatory myopathies led us to hypothesize that IVIG might induce autophagy in immune cells and hence represents a novel mechanism of action of IVIG in autoimmune and inflammatory conditions. In fact, our data show that IVIG therapy induces autophagy in immune cells and is associated with inhibition of mTOR phosphorylation.

IVIG has been demonstrated to modulate the functions of both innate and adaptive immune cells. Thus, IVIG suppresses the activation of various innate cells such as DCs, macrophages, and monocytes, and the secretion of inflammatory cytokines while enhancing anti-inflammatory mediators like IL-10 and IL-1RA^21,48–52^. The present data demonstrate that IVIG exerts autophagy in several inflammatory innate cells like monocytes, DCs and M1 macrophages but not in the cells associated with Th2 immune response like M2 macrophages. The inability of IVIG to induce autophagy in M2 macrophages might not be due to differences in the endocytosis activity of these innate cells. In fact, previous data have shown that M2 macrophages have more endocytic activity compared to M1 macrophages^53^. On the other hand, cytokines that are produced by M2 macrophages such as IL-13 and IL-10 are known to inhibit autophagy^41,42^. Also, autophagy machinery is intact in M2 macrophages as starvation condition was able to induce autophagy in M2 macrophages (Fig. 2d). All these evidences thus suggest that lack of induction of autophagy by IVIG in M2 macrophages is likely due to the cytokines produced by M2 macrophages.

In view of diverse role of autophagy in the regulation of inflammation and autoimmune responses, we propose that autophagy is central to the anti-inflammatory actions of IVIG. In support of this argument, we found that the ability of IVIG to suppress the production of inflammatory cytokines is compromised when autophagy was inhibited. Although induction of autophagy by IVIG was higher at concentrations corresponding to high-dose therapy in autoimmune diseases, autophagy was significantly induced by IVIG even at concentrations corresponding to the circulating levels of IgG (10 mg) in healthy individuals. As IVIG is a pool of IgG from the plasma of healthy individuals, these results thus point towards a role for circulating normal IgG in the maintenance of immune homeostasis.

While mTOR acts as negative regulator of autophagy, beclin-1 and several kinases like class III PI3K, p38 MAPK and AMPK are critical for initiating autophagy although several studies also suggest that p38 MAPK has dual role in autophagy^54,55^. In this regard, the role of AMPK is of particular importance as phosphorylation of AMPK under the conditions of low cellular energy and essential amino acid deprivation directly antagonizes mTOR leading to initiation of autophagy^56,57^. Emerging evidences imply that AMPK is a key modulator of immune response. AMPK could reduce the severity of inflammation and tissue damage in various models of autoimmune diseases^58,59^. In this study, we show that IVIG-induced autophagy is associated with induction and activation of AMPK, beclin-1, class III PI3K and p38 MAPK. It is not clear at this stage whether inhibition of mTOR by IVIG is mediated via AMPK or due to direct suppressive effects on mTOR. However, abrogation of IVIG-induced autophagy upon inhibition of AMPK implies that mTOR inhibition by IVIG was not due to its direct effect on this molecule rather via AMPK-mediated process.

Exploration of mechanisms by which IVIG induces autophagy has revealed that F(ab′)_2_ fragments induce autophagy similar to intact IgG while desialylation of IgG had no repercussion. This indicates that glycosylation patterns of Fc-fragments including sialylation^26,60^ have no major role in IVIG-induced autophagy. Indeed, several F(ab′)_2_ fragments-mediated functions of IVIG have been reported like neutralization of C3a and C5a anaphylatoxins and pathogenic autoantibodies, regulation of human DCs and granulocyte functions, inhibition of phagocytosis, expansion of human regulatory T cells and inhibition of Th17 responses^61–68^. However, we found that IVIG might not induce autophagy by signaling via cell surface receptors, as inhibition of endocytosis of IgG via cytochalasin D that inhibits phagocytosis and macropinocytosis abrogated IVIG-induced autophagy. Several reports have demonstrated that innate immune cells internalize IVIG^28,34,35^. As equivalent amount of HSA failed to induce autophagy, this rules out the possibility that IVIG-induced autophagy is due to the stress caused by accumulation of high amounts of proteins in the endosomes. As IVIG has been demonstrated to interact with various cytoplasmic antigens^69^, this suggests that IVIG might induce autophagy either by modifying the autophagy cargo or interacting with molecules that promote autophagy. Dependency of IVIG on F(ab′)_2_ fragments to induce autophagy also point towards this possibility.

Several lines of evidence show that IVIG is an effective therapy for inflammatory myopathies including dermatomyositis and polymyositis^17–20,70^. IVIG therapy response in inflammatory myopathy patients is associated with the modulation of various populations of immune cells and inflammatory mediators^68,71–76^. Our current data also demonstrate that IVIG therapy results in the induction of autophagy in PBMCs and downregulation of several inflammatory cytokines in the circulation including IL-8, TNF-α, IL-1β and IL-17A. However, induction of autophagy might not be beneficial in all the pathologies. In inclusion body myositis (IBM), increased maturation of autophagosomes has been observed in muscle fibers but lysosomal proteolytic activity was decreased resulting in the enhanced accumulation of misfolded proteins^13^. Other muscular disorders like IBM associated with Paget’s disease of the bone, fronto-temporal dementia and amyotrophic lateral sclerosis (IBMPFD/ALS), and X-linked myopathy with excessive autophagy (XMEA) also show defective autophagosome maturation and enhanced autophagosome biogenesis^77,78^. Although it is not known whether IVIG could induce autophagy in the muscle fibers similar to immune cells demonstrated here, these reports provide a pointer on why IVIG has only limited therapeutic benefits in IBM patients.

To conclude, our study demonstrates that IVIG therapy induces autophagy in patients with inflammatory myopathies. Further investigations revealed that IVIG-induced autophagy is restricted to inflammatory cells like monocytes, DCs and M1 macrophages but not in cells associated with Th2 immune response like M2 macrophages. Mechanistically, IVIG induces autophagy by F(ab′)_2_-dependent process via activation of p38 MAPK, AMPK and class III PI3K and this requires endocytosis of IgG. Autophagy process constitutes an integral arm of anti-inflammatory mechanisms of IVIG. Our data thus provide insight on how circulating normal immunoglobulin maintains immune homeostasis and explains in part the mechanism by which IVIG therapy benefits patients with autoimmune and inflammatory diseases.

## Materials and methods

### Reagents and antibodies

IFN-γ, M-CSF and IL-13 were from R&D Systems (Lille, France). CD14 and CD4 MicroBeads, GM-CSF and IL-4 were from Miltenyi Biotec (Paris, France). Rabbit MAbs to p-p38MAPK (Thr180/Tyr182, Clone 12FB), PI3K class III (Clone D9A5), Beclin-1 (Clone D40C5), β-actin (Clone 13E5, HRP-conjugated), GAPDH (Clone 14C10, HRP-conjugated); affinity purified antibodies to p-mTOR (Ser2448, #2971), mTOR (#2972), LC3B (#2775), p-AMPK (Thr172, #2531) AMPK (#2532) p38MAPK (#9212), HRP-linked anti-rabbit IgG (#7074) were from Cell Signaling Technology (Ozyme, Saint Quentin Yvelines, France). Plasma-derived HSA was from Laboratoire Française de Biotechnologies (Les Ulis, France) and *E. coli* 055:B5 lipopolysaccharide was purchased from Sigma-Aldrich Chimie S.a.r.l (St. Quentin Fallavier, France).

### Therapeutic IVIG

Sandoglobulin® (CSL Behring, Bern, Switzerland) was used throughout the study. IVIG was dialyzed for 18 h against large volumes of phosphate-buffered saline (PBS) followed by RPMI-1640 at 4 °C f. F(ab′)_2_ fragments were generated by digesting IVIG with pepsin (Sigma-Aldrich) at 50:1 ratio for 18 h in 0.2 M sodium acetate buffer (pH 4.1). F(ab′)_2_ fragments were extensively dialyzed against sterile PBS followed by RPMI-1640 medium at 4 °C and filtered through 0.22 μm membrane. Purity of F(ab′)_2_ fragments were verified by SDS-PAGE and Coomassie blue staining.

For desialylation of IgG, IVIG (Hizentra®, CSL Behring) was treated with recombinant neuraminidase (New England BioLabs, USA) as previously described^29^. By lectin blotting and reversed phase -HPLC, we confirmed the desialylation of IgG.

### Purification of peripheral blood mononuclear cells and monocytes

Peripheral blood mononuclear cells were obtained from the buffy bags of healthy donors by Ficoll density gradient centrifugation. Buffy bags were purchased from Centre Necker-Cabanel, Etablissement Français du Sang, Paris, France. Ethical committee permission was obtained for the use of buffy bags of healthy donors (Institut National de la Santé et de la Recherche-EFS ethical committee convention 15/EFS/012). Monocytes were isolated from the PBMCs by positive selection using human CD14 MicroBeads. The cell purity was more than 97%.

### Generation of DCs, M1 and M2 macrophages

DCs were generated from monocytes by differentiating them in the presence of GM-CSF (1000 IU/10^6^ cells) and IL-4 (500 IU/10^6^ cells) for 6 days^79^.

To obtain macrophages, monocytes were cultured in the presence of M-CSF (200 ng/10^6^ cells) for 6 days. These macrophages were further polarized into either M1 or M2 macrophages by culturing in the presence of lipopolysaccharide (200 ng/10^6^ cells) and IFN-γ (40 ng/10^6^ cells), and IL-4 (40 ng/10^6^ cells) and IL-13 (40 ng/10^6^ cells) respectively for 3 additional days.

### Culturing of immune cells

PBMCs, peripheral blood monocytes, monocyte-derived DCs or macrophages (M1 and M2) were cultured in RPMI-1640 containing 10% fetal calf serum (0.5 million cells/ml) either alone or with various concentrations of IVIG preparations (10 and 25 mg/ml; native or desialylated IVIG) or equimolar concentrations of F(ab′)_2_ fragments of IVIG for 24 h unless otherwise stated. All cells were treated with bafilomycin (50 nM) for last 45 min. The equimolar concentration of HSA (0.15 mM) was used as an irrelevant protein control. Autophagy was analyzed by measuring the LC3-II levels by western blots or by immunofluorescence.

As a positive control for autophagy, immune cells were cultured in Hank’s Balanced Salt Solution for 4 h to induce autophagy by starvation or were treated with 100 nM rapamycin (Calbiochem, Merck Chimie SAS, Fontenay sous Bois, France) for 24 h.

For visualizing the autophagic organelles in IVIG-treated DCs by transmission electron microscopy, monocyte-derived DCs were cultured for 24 h either with lipopolysaccharide alone or with IVIG (25 mg/ml) added 30 min post-lipopolysaccharide stimulation. DCs were washed with PBS and then fixed with 2.5% glutaraldehyde (in 0.1 M PBS, pH 7.4) for 30 min. Fixed DCs were processed for transmission electron microscopy by standard procedure^80^ and cells were observed with a Zeiss EM 10 electron microscope (Carl Zeiss Microscopy GmbH, Jena, Germany)

For measuring the autophagy flux, PBMCs from healthy donors were cultured with IVIG (25 mg/ml) for 0, 18, or 24 h. At the end of treatment, cells were treated with or without bafilomycin (Baf) for 45 min. Autophagy flux (∆LC3-II) was quantified by subtracting the band intensity of LC3-II in the presence of Baf from that of absence of Baf by using the formula:

$$\Delta {\mathrm {LC3}}{\hbox{-}}{\mathrm {II}} = \left[{\mathrm {LC3}}{\hbox{-}}{\mathrm {II}}( + {\mathrm {Baf}})\right] - \left[{\mathrm {LC3}}{\hbox{-}}{\mathrm {II}}( - {\mathrm {Baf}})\right]$$

### Treatment of monocytes with pharmaceutical inhibitors

Different pharmaceutical inhibitors, i.e., 3-methyladenine (3MA, for PI3K class III), SB202190 (for p38MAPK) and compound C (Comp C, for AMPK) were used in this study. Cytochalasin D was used to block endocytosis. The concentration of inhibitors was chosen after careful titration experiments assessing the viability of the monocytes. Monocytes were pretreated with 3MA (1 and 5 mM, for western blot and cytokine analyses respectively), SB202190 (10 μM), compound C (5 μM) and cytochalasin D (10 μM) for 1 h with inhibitors followed by culture with IVIG for 24 h. The cells were then processed for immunoblot.

### Inflammatory myopathy patients

Heparinized blood samples of patients with inflammatory myopathies were obtained before and 24−48 h following initiation of IVIG (2 g/kg) therapy. Informed consent was obtained from the patients. PBMCs were isolated by Ficoll density gradient centrifugation and processed for immunoblot. We analyzed five myopathy patients (two immune-mediated necrotizing myopathy patients, two dermatomyositis patients and one antisynthetase syndrome patient) with a median age of 48 years (ranging from 38 to 64) and include two men (Supplementary Table S1). The study was approved by CPP-Ile-de-France VI, Groupe Hospitalier Pitié-Salpêtrière, Paris. IgG levels in the plasma were measured by immunonephelometry (Cobas 6000 Roche Diagnostics).

### Measurement of inflammatory cytokines

Lipopolysaccharide (100 ng/0.5 million cells/ml)-stimulated DCs were treated with 3MA (5 mM) for 1 h followed by culture with IVIG (25 mg/ml) for 24 h. Amounts of DC-cytokines (IL-8, IL-6 and IL-1β) in the cell-free supernatants and cytokines in the plasma of inflammatory myopathy patients (IL-8, TNF-α, IL-1β and IL-17A) were analyzed by ELISA (ELISA Ready-SET-Go, eBioscience, Paris, France).

### Immunoblotting analysis

Treated cells were pelleted and lysed in RIPA buffer (Sigma) containing protease and phosphatase inhibitors (Roche Biotech) on ice for 30 min. Whole-cell lysates were collected. Proteins were quantified and equal amount of proteins from each sample was separated on SDS-PAGE. The proteins were then transferred onto polyvinylidene fluoride membranes by a wet western blotting method. The membrane was blocked using 5% BSA in TBST (20 mM Tris-HCl [pH 7.4], 137 mM NaCl, and 0.1% Tween 20) for 60 min. The blots were incubated overnight at 4 °C with primary antibody diluted in TBST-5 % bovine serum albumin (BSA) followed by incubation with HRP-conjugated anti-rabbit IgG secondary antibody for 2 h. After washing in TBST, blots were developed with SuperSignal^TM^ WestDura Extended Duration substrate (ThermoFisher Scientific, Courtaboeuf, France) according to the manufacturer’s instructions. β-actin or GAPDH was used as loading control. The western blot images were analyzed by myImageAnalysis software v 2.0 (ThermoFisher Scientific). Full western blot images are presented in Supplementary Fig. S1.

### Cell line

THP-1, a pro-monocytic cell line (obtained from the National Center for Cell Sciences, Pune, India) was maintained in RPMI-1640 medium containing 10% fetal bovine serum (FBS). Cells (0.5 million cells/ml) were treated with IVIG (25 mg/ml) or equimolar concentrations of HSA for 24 h. In case of inhibitor treatment, cells were pretreated with inhibitors for 1 h followed by culture with IVIG for 24 h. Cells were treated with bafilomycin for last 45 min.

### Immunofluorescence

Treated THP-1 cells were collected, fixed with 4% paraformaldehyde and washed with PBS. After blocking with blocking buffer (1× PBS, 5% FBS and 0.3% Triton X-100), cells were stained at 4 °C with polyclonal affinity purified rabbit anti-MAP1LC3I/II antibody (#4108, Cell Signaling Technology) diluted in antibody dilution buffer (1× PBS, 1% BSA and 0.3% Triton X-100) for overnight. Cells were washed thoroughly three times and incubated with goat anti-rabbit IgG (H + L), F(ab′)_2_ fragment (Alexa Fluor® 488 conjugate, Cell Signaling Technology, #4412) for 2 h. The nuclei were stained with 4′,6-Diamidino-2-phenylindole dihydrochloride (DAPI, Sigma-Aldrich) for 10 min. Cells were spread on coverslip and mounted with glycerol as a medium. Confocal images were taken using Zeiss LSM 710 meta confocal laser scanning microscope using a Plan-Achromat ×63/1.4 oil DIC objective (both from Carl Zeiss). Images were analyzed using ZEN 2009 software.

### Statistical analysis

As detailed in the figure legends, each experiment was repeated independently 3−7 times using different donors, except for autophagy flux that was repeated twice. Sample size was chosen based on the previous experience with western blot and biological assays, and literature survey. We did not use any specific test to ensure adequate power. The data were presented as mean ± standard error of the mean (SEM) unless otherwise specified. Randomization and blinding were not used in the study. Statistical analyses were performed by two-way nonparametric Mann–Whitney test or one-way ANOVA (Dunnett’s or Tukey’s multiple comparison tests) as indicated in the figure legends using Prism 6 software. Values were deliberated statistically significant with *P < 0.05, **P < 0.01, ***P < 0.001, and ****P < 0.0001.

## Supplementary information

Supplementary Table legend

Supplementary Table S1

Supplementary Figure legend

Supplementary Fig S1
