# Supplementary material for: Intravenous immunoglobulin mediates anti-inflammatory effects in peripheral blood mononuclear cells by inducing autophagy
Source: Cell Death Dis. 2020 Jan 23;11(1):50. doi: 10.1038/s41419-020-2249-y (PMC6978335; doi:10.1038/s41419-020-2249-y)
Supplement: Supplementary file 2 — Supplementary Table S1 [file 41419_2020_2249_MOESM2_ESM.docx]

| Patient | Age  (Years) | Gender | Pathology | IVIG dose | Pre-IVIG  IgG in the plasma  (g/L) | Post-IVIG IgG in the plasma  (g/L) | Timing of post-IVIG blood collection |
| --- | --- | --- | --- | --- | --- | --- | --- |
| 1 | 53 | F | Antisynthetase syndrome, anti-Jo1 antibodies | 2 g/kg | 14.7 | 42.6 | 36 hours |
| 2 | 40 | F | Immune-mediated necrotizing myopathy, anti-HMGCR antibodies | 2 g/kg | 16.6 | 56.9 | 36 hours |
| 3 | 64 | M | Dermatomyositis, no antibodies | 2 g/kg | 15.4 | 55.4 | 48 hours |
| 4 | 38 | M | Dermatomyositis, anti-NXP2 antibodies | 2 g/kg | 16.3 | 46.9 | 48 hours |
| 5 | 48 | F | Immune-mediated necrotizing myopathy, anti-SRP antibodies | 2 g/kg | 17.1 | 48.9 | 24 hours |

**Supplementary Table S1**. **Summary of data for myopathy patients**

F: Female

M: Male

HMGCR: 3-hydroxy-3-methylglutaryl-coenzyme A reductase

NXP2: antinuclear matrix protein 2

SRP: signal recognition particle
