# Supplementary material for: Intravenous immunoglobulin mediates anti-inflammatory effects in peripheral blood mononuclear cells by inducing autophagy
Source: Cell Death Dis. 2020 Jan 23;11(1):50. doi: 10.1038/s41419-020-2249-y (PMC6978335; doi:10.1038/s41419-020-2249-y)
Supplement: Supplementary file 3 — Supplementary Figure legend [file 41419_2020_2249_MOESM3_ESM.docx]

**Supplementary Fig S1**. **Full western blot images**.

Western blot images shown in the article are highlighted by boxes. Please note that irrelevant bands in the supplementary figures are represented by *.
