# Supplementary figures and images for: Intravenous immunoglobulin mediates anti-inflammatory effects in peripheral blood mononuclear cells by inducing autophagy
Source: Cell Death Dis. 2020 Jan 23;11(1):50. doi: 10.1038/s41419-020-2249-y (PMC6978335; doi:10.1038/s41419-020-2249-y)

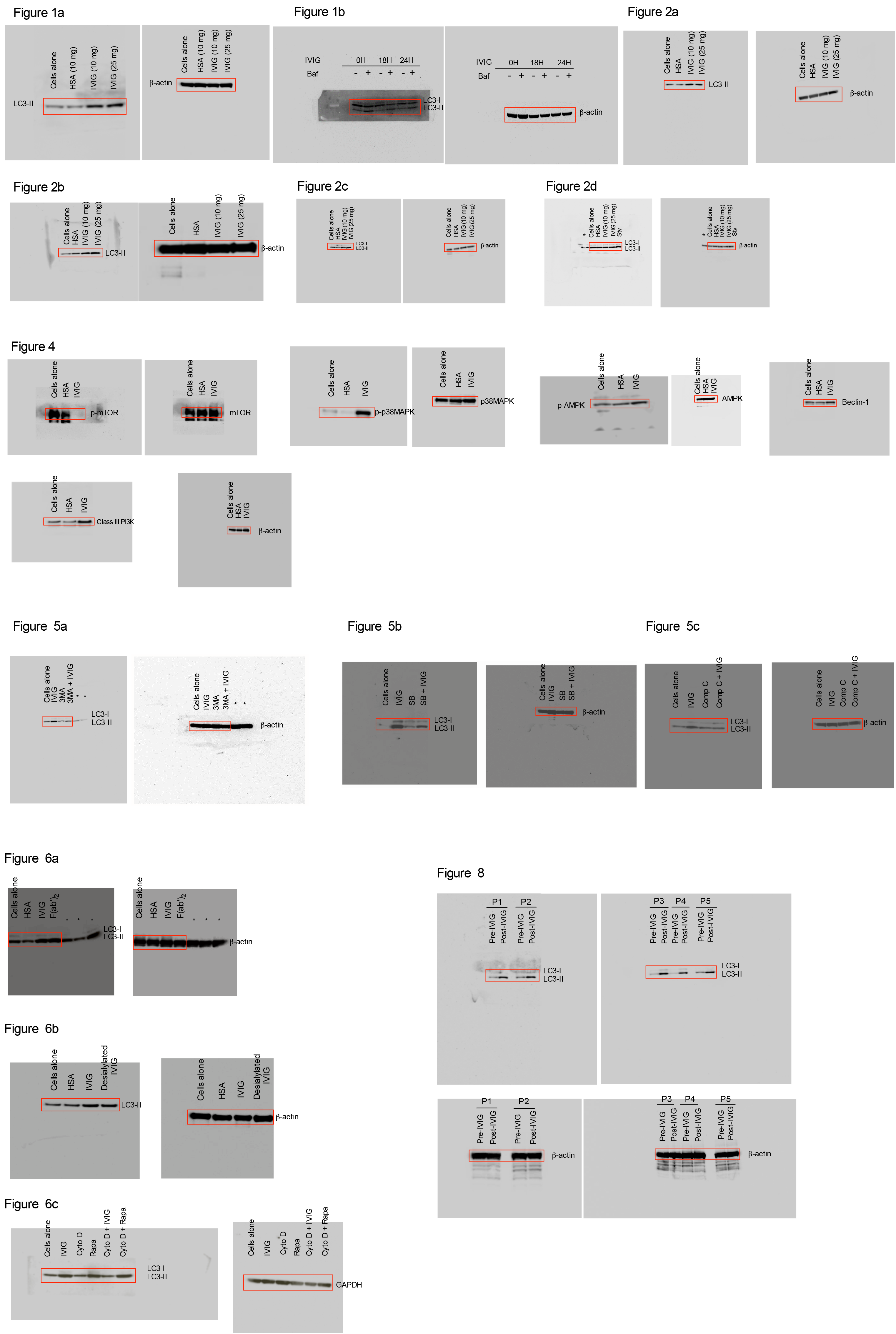

Supplement: Supplementary file 4 — Supplementary Fig S1 [file 41419_2020_2249_MOESM4_ESM.tif]
